# Microbial glycoconjugates in organic pollutant bioremediation: recent advances and applications

**DOI:** 10.1186/s12934-021-01556-9

**Published:** 2021-03-18

**Authors:** Pankaj Bhatt, Amit Verma, Saurabh Gangola, Geeta Bhandari, Shaohua Chen

**Affiliations:** 1 State Key Laboratory for Conservation and Utilization of Subtropical Agro-bioresources, Guangdong Laboratory for Lingnan Modern Agriculture, Integrative Microbiology Research Centre, South China Agricultural University, Guangzhou, 510642 China; 2 Guangdong Province Key Laboratory of Microbial Signals and Disease Control, South China Agricultural University, Guangzhou, 510642 China; 3 Department of Biochemistry, College of Basic Science and Humanities, SD Agricultural University, Gujarat, 385506 India; 4 School of Agriculture, Graphic Era Hill University, Bhimtal Campus, Dehradun, Uttarakhand 248002 India; 5 Department of Biotechnology, Sardar Bhagwan Singh University, Dehradun, Uttarakhand 248161 India

**Keywords:** Glycoconjugates, Bioremediation, Biosurfactants, Organic pollutants, Biofilm

## Abstract

The large-scale application of organic pollutants (OPs) has contaminated the air, soil, and water. Persistent OPs enter the food supply chain and create several hazardous effects on living systems. Thus, there is a need to manage the environmental levels of these toxicants. Microbial glycoconjugates pave the way for the enhanced degradation of these toxic pollutants from the environment. Microbial glycoconjugates increase the bioavailability of these OPs by reducing surface tension and creating a solvent interface. To date, very little emphasis has been given to the scope of glycoconjugates in the biodegradation of OPs. Glycoconjugates create a bridge between microbes and OPs, which helps to accelerate degradation through microbial metabolism. This review provides an in-depth overview of glycoconjugates, their role in biofilm formation, and their applications in the bioremediation of OP-contaminated environments.

## Introduction

**Fig. 1:**
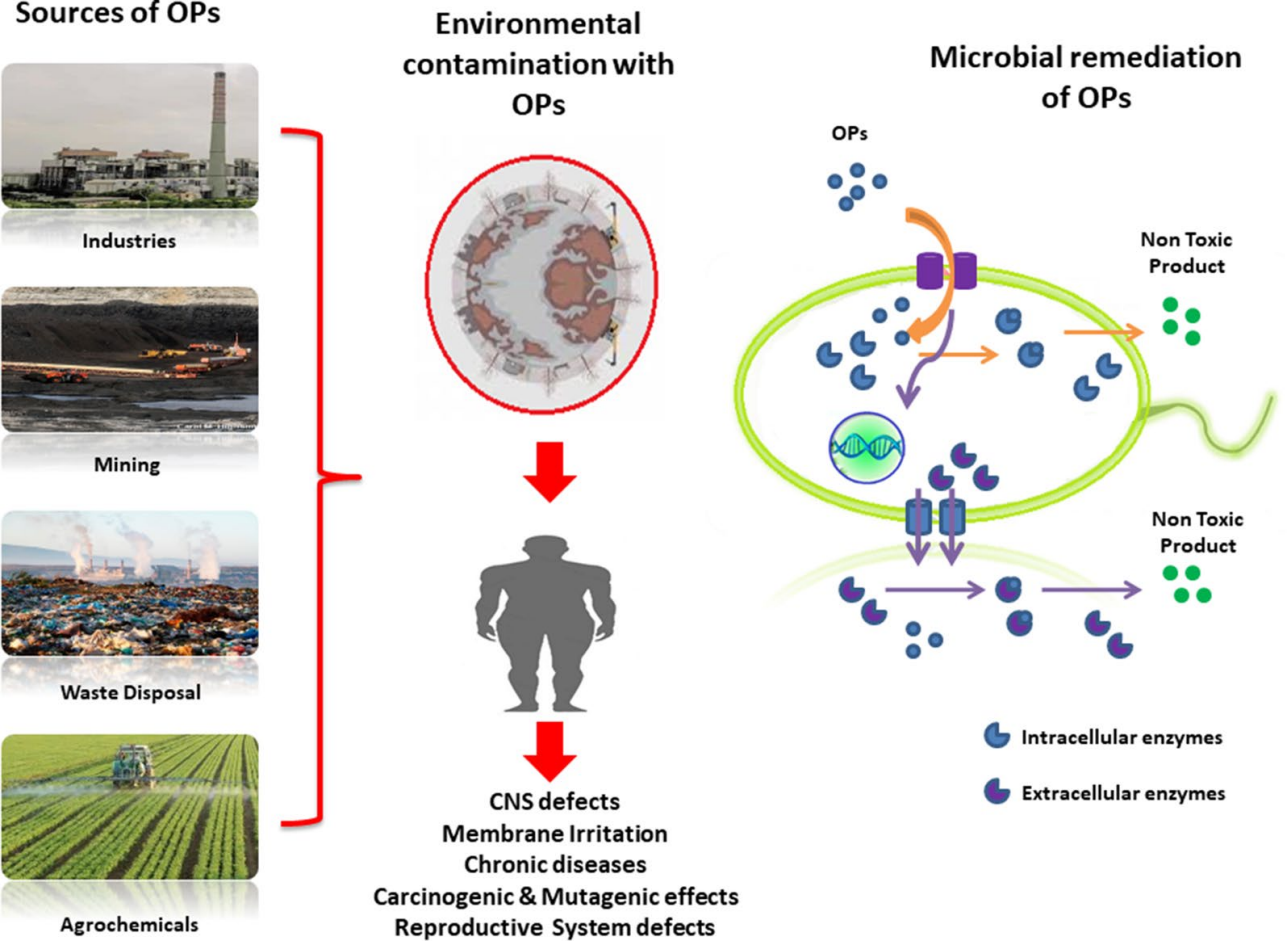
Microbial remediation of organic pollutants (OPs): sources, adverse effects and micro-remediation mechanisms

Organic pollutants (OPs) are used in large quantities in the industrial and agricultural sectors [1]. The rapid industrialization and anthropogenic activities of the present era have increased environmental contamination with various OPs, including compounds like chloroform, benzene, carbon tetrachloride, paints, gasoline, adhesives, plastic compounds, chlorohydrocarbons (CHCs), and pesticides [2]. OPs are presently found in the air, soil, and water and have various adverse effects on living systems, including the flora and fauna present in the ecosystem [3]. These OPs are also reported to be responsible for various toxic effects in humans, including adverse carcinogenic, mutagenic, and teratogenic effects. Thus, remediation strategies for these OPs are essential in the present scenario (Fig. 1). The remediation of OPs usually uses physical and chemical techniques such as soil washing, pumping, aeration, oxidation, incineration, etc. [4]. However, these methods have many disadvantages and usually result in secondary environmental contamination; they are also uneconomical to use. The secondary contaminants are not emitted directly from the source they formed due to degradation reactions of the main pollutants. Therefore, bioremediation strategies utilizing living systems are the only hope for the eco-friendly management of these OPs.

Microbial bioremediation (MB) is usually considered one of the best methods for the treatment of environmental contamination. The rich diversity of metabolizing enzymes participated in the bioremediation processes [3]. The MB of contaminants is possible through enzymatic reactions, which produce different intermediate metabolites through metabolic pathways. Although single microbial cultures have been used as potent contaminant degraders in recent decades, but mixed cultures perform better in environments [5]. Environmental contamination with OPs can be managed by utilizing microbial metabolic processes that degrade these OPs into non-toxic metabolites in an economical, eco-friendly, and efficient manner [6]. Thus, researchers are involved in the study of microbial biodegradation mechanisms related to OPs to develop low cost and simple techniques for the management of these pollutants. OPs are metabolized by microbial cells using both aerobic and anaerobic metabolism. Anaerobic metabolism is one of the most preferred methods in bioremediation, especially for chlorinated OPs. However, sometimes OPs involve the production of much more toxic compounds, such as trichloroethylene (TCE). Microbial degradation via anaerobic mechanisms results in the production of dichloroethylene (DCE) and vinyl chlorides (VCs), which have higher environmental toxicity than their parent compound, TCE [2]. Thus, at times, aerobes are the best choice for OP bioremediation due to presence of various catabolite enzymes with broad specificity to degrade different types of OPs. These aerobes consist of various oxygenases that play a significant role in the degradation of pollutants from contaminated sites. For example, *Pseudomonas* sp. has oxygenases that can metabolize TCE along with the associated DCE and VCs into CO_2_ and Cl^-^, where both the final products are non-toxic [4]. However, the efficient degradation of OPs rests in understanding its transportation inside the microbial cell and its assimilation. Studies indicate that microbial glycolipids and other glycoconjugates play a very important role in the mechanism of transport of these OPs across microbial membranes [7]. These microbial glycol compounds act as emulsifiers and are called “biosurfactants”, which are located either inside the cell or secreted outside and help in the bioremediation mechanism [8]. This gave rise to the term “microbial glycobiotechnology” (MG), which involves a wide array of methods, with the main goal of decontaminating different types of pollutants.

MG utilizes natural microbial resources for the transformation of the contaminated environment to a safe native natural form. MG involves the microbial production of carbohydrate polymeric compounds with novel applications in the field of bioremediation and waste management. Studies proved that biosurfactant production has a direct correlation to OP degradation. Thus, MG is gaining importance for the management of OPs in the environment [9]. MG interacts with proteins and metabolites and facilitates the degradation of OPs [10]. This review presents an overview of recent advances in MG and its specific applications in the bioremediation of different types of OPs.

### Microbial glycoconjugates: types and application in bioremediation

**Fig. 2:**
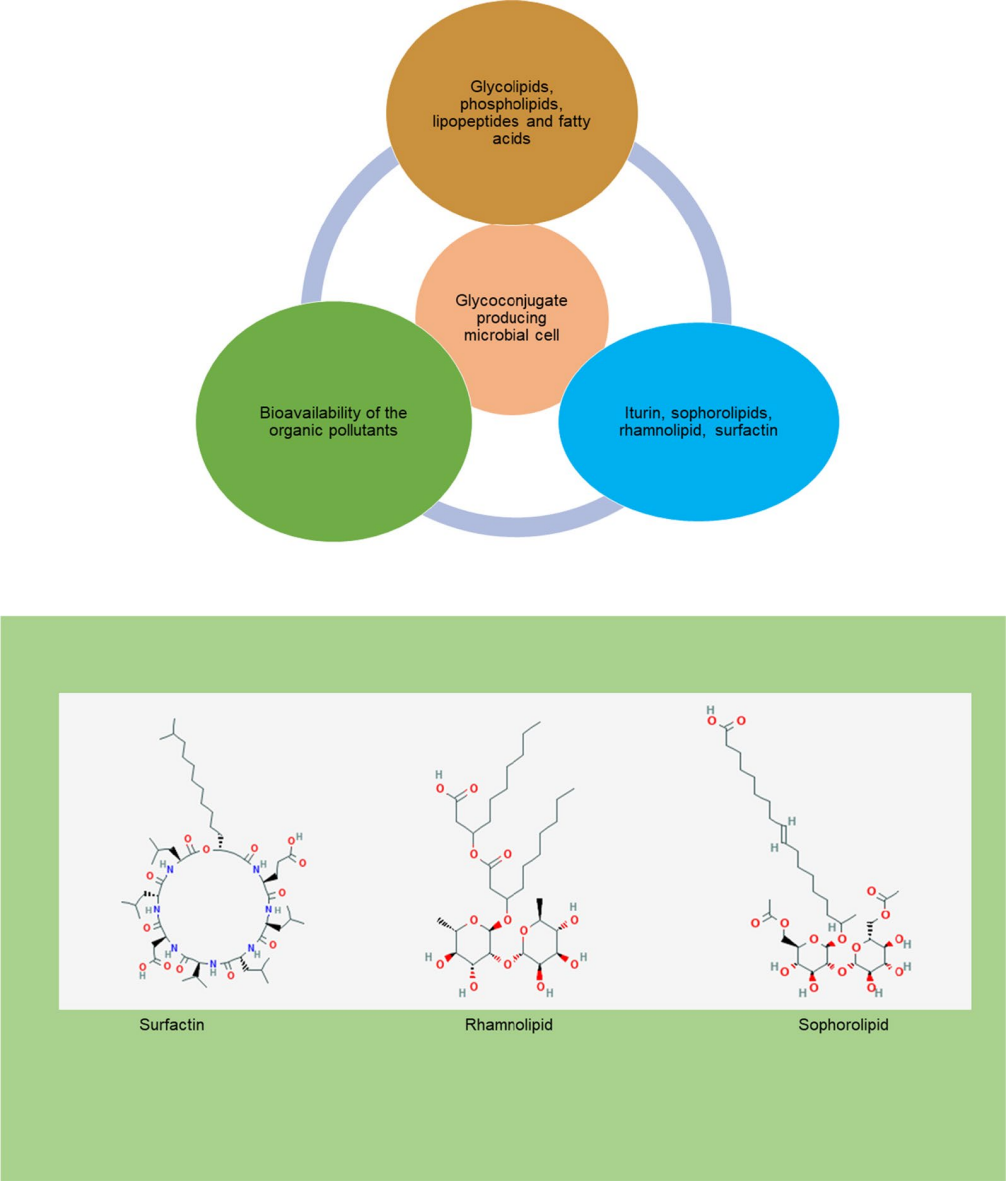
Microbial glycoconjugates in the bioremediation of organic pollutants (OPs)

Glycobiotechnology, involves the transfer of the basic knowledge structure and functional relationship of glycoconjugates to practice-related synthetic and applied producers [11]. The term “glycoconjugate” indicates the combination of glycoproteins and glycolipids. Microbial strains are able to produce glycoconjugates and facilitate their metabolism in various ways, such as via the producers of these molecules, uptake of the desirable pollutants, and other substrates (Table 1). Glycoconjugates are an integral part of the bacterial cell membrane, which consists of special types, viz., surface molecules (lipopolysaccharides, capsular polysaccharides, lipo-oligosaccharides, and glycoproteins), cell-wall polymers, and secreted exopolysaccharides [12] (Fig. 2). In addition to this, microbial strains produce extracellular glycoconjugates such as rhamnolipids, sophorolipids and exopolysaccharides, glycoproteins, and glycol-lipopeptides. These glycoconjugates play a crucial role in the bioremediation of the OPs [13].

**Table 1:** Glycoconjugates in the bioremediation of organic pollutants (OPs)

Microbial strains	Glycoconjugates	Organic pollutants	Mode of action	References
*Acinetobacter* sp. Y1	Methyl hexadcanoate, methyl octadecanoate	Petroleum hydrocarbon	Reduce surface tension of water, showed strong tolerance with pH, temperature, salinity	[14]
*Pseudomonas*, *Rhodococcus*	Biosurfactants	Cypermethrin	Emulsion reaction	[15]
*Achromobacter* sp. A-8	Biosurfactants	Crude oil	Reduce surface tension	[16]
*Acinetobacter baumannii* BJ5	Glycolipid biosurfactant	Pyrene	Growth linked production	[17]
*Burkholderia cenocepacia* BSP3	Glucolipid	Methyl parathion, ethyl parathion, trifluralin	Critical micelle formation (CMC) and reducing surface tension	[18]
*Pseudomonas aeruginosa* WH-1	Biosurfactants	Hexachlorocyclohexane (HCH)	Lower the emulsification with HCH	[19]
*Pseudomonas* sp.	Rhamnolipids	Chlorpyrifos	Increase the aqueous partition and chlorpyrifos degradation	[20]
*Bacillus subtilis* MTCC 1427	Biosurfactants	Endosulfan	Increase bioavailability of endosulfan	[21]
*Pseudomonas aeruginosa* B1, *P. fluorescens* B5, *P. stutzeri* B11 and *P. putida* B15	Exopolysaccharides (EPS)	2,4-D, benzene, toluene, xylene and gasoline	Organic pollutants affect EPS production	[22]
*Penicillium simplicissimum*	Tea saponin, rhamnolipid	Phenol	CMC, reduce surface tension and increase laccase production	[23]
*Pseudomonas aeruginosa* CH7	Rhamnolipid	*β*-Cypermethrin	Rhamnolipid promote the dissolution, absorption, adsorption	[24]
*Candia*, *Pseudomonas*, *Deinococcus*, *Nocardiopsis*, *Serratia*	Rhamnolipids, trehalolipids, mannosylerythritol lipids, cellobiose lipids	Organic pollutants	Bioremediation of the organic pollutants	[25]
*Pseudomonas, Bacillus*, *Candida*	Rhamnolipid	Oil spill	Reduce interfacial tension, disperse oil particles	[26]
*Pseudomonas aeruginosa*, *Rhodococcus* sp., *Bacillus licheniformis*, *Serratia marcescens*, *P. flourescens*, *B. subtilis*	Rhamnolipid, trehalolipid, sophorolipid, peptide lipid, serrawetin, visconsin, surfactin, emulsan, liposan	Oil pollution	Enhanced degradation	[27]
*Serratia marcescens* UCP 1549	Lipoprotein, carbohydrate	Organic pollutants	Agricultural and marine bioremediation	[28]
*Bacillus subtilis* B20	Biosurfactants	Oil rock	Reduced surface and interfacial tension	[29]
*Paenibacillus* sp. D9	Lipopeptide biosurfactant	Hydrocarbons	Enhanced biodegradation of hydrophobic pollutants	[30]
*Bacillus*, *Rhodococcus*, *Actinomycetes*, *Pseudomonas*	Lipopeptide, glycolipid, sophorolipds	Organic pollutants	Reduce surface tension with higher degradation	[31]
*Bacillus algicola*, *Rhodococcus soli*, *Isoptericola chiayiensis*, *Pseudoalteromonas agarivorans*	Rhamnolipids	Crude oil	Low surface tension	[32]

Microorganisms produce glycoconjugates with biosurfactant properties during the stationary phase of the microbial growth cycle [33, 34]. Glycoconjugates are amphiphilic compounds synthesized onto the cell surface of the microorganism [35]. These molecules contain hydrophilic and hydrophobic moieties that reduce the surface and interfacial tension. Glycoconjugates can have diverse structures, such as glycoproteins, glycopeptides, peptidoglycans, glycolipids, lipopolysaccharides, and glycosides. The production of the glycoconjugates depends on the producer microorganism, nutritional sources such as carbon and nitrogen, trace elements, and the physicochemical conditions for production. Recently, glycoconjugate rhamnolipids have been the most commonly used in industrial and environmental applications [35, 36]. The glycolipid rhamnolipid is well studied in the *Pseudomonas* and *Burkholderia* species [36]. *Pseudomonas aeruginosa* is considered as the top rhamnolipid producer at over 100 g·L^− 1^. In a liquid culture, *Pseudomonas aeruginosa* produces two types of rhamnolipids referred to as mono and dirhamnolipid [35]. These molecules are synthesized by two enzyme-specific rhamnosyl transfer reactions. The enzyme that catalyzes these reactions is called rhamnosyltransferase [37, 38]. The hydrophobic and hydrophilic parts of the rhamnolipid are synthesized by different biosynthetic reactions in the microbial strains. After their synthesis, both of the portions are linked to each other, forming monorhamnolipids and dirhamnolipids. Yeasts are also reported to produce glycoconjugates such as sophorolipids, mannosylerythritol, cellobiose, and trehalose lipids. These have been explored for their greater potential in the bioremediation of polluted sites [39]. The enhanced bioremediation of pyrene and tetracycline in soil was investigated with the addition of sophorolipid [40].

Hydrophobic pollutants require desorption from the soil and water environment before microbial metabolism. Mineralization of OPs is governed by desorption from the soil. The application of glycoconjugates as biosurfactants for the bioremediation of environmental OPs is also well established; they play a direct role in the desorption of pollutants [41]. In the first step, these glycoconjugates interact with less soluble OPs and improve their transfer into the soil matrix and their subsequent removal [42]. In the second step, glycoconjugates act as a bridge between the microbial strains and soil, due to which the bioavailability of the pollutants increases [43, 44]. The increased concentrations of these surface-active glycoconjugate compounds help in the attachment of microbial cells to pollutants [45]. Biosurfactants increase the surface areas of hydrophobic pollutants through which their solubility increases in the soil and water environment. The use of biosurfactants for the biodegradation of pesticides has gained attention in recent years. Previous reports supported the role of biosurfactants in the bioremediation of hydrocarbon and pesticide-contaminated soil. These reports favor pesticide degradation using glycoconjugated biosurfactant usually synthesized from bacterial species viz., *B. pumilus*, *B. mojavensis*, *B. licheniformis* and *B. amyloliquifaciens* [46]. Biosurfactants of *Lactobacillus pentosus* degrade octane efficiently [47]. In a study, *Burkholderia* species isolated from an oil-contaminated area was able to produce biosurfactant, that plays a critical role in pesticide degradation [18, 48]. Biosurfactants that degrade naturally are ideally suitable for the removal of organic pollutants from the environment and considered ecofriendly to nature [49]. Previous studies indicated that the efficiency of OP degradation was improved in the presence of microbial glycoconjugates. Stimulation in the degradation of OPs was mainly due to the action of the biosurfactants. Enhancement in the degradation of octane was due to the biosurfactants production using *Lactobacillus pentosus* [47]. In addition to mobilization, glycoconjugated biosurfactants increase the degradation rate via other mechanisms [50]. An axenic culture of *Pseudomonas putida* DOT-T1E produced a rhamnolipid that facilitated the bioremediation of chlorinated phenols. The logic behind this mechanism involves entrapment of the chlorophenol in the biosurfactant micelles and the hydrophobic relationship between these two types of compounds [51, 52]. Similarly, *Actinobacteria* produced biosurfactants that enhanced the rate of xenobiotics bioremediation [53]. Rhamnolipids were found to be adequate in the bioremediation of carbendazim with *Rhodococcus* sp. D-1 [54]. The rhamnolipid affected carbendazim degradation in a concentration-dependent manner with maximum bioremediation efficiency. It facilitated carbendazim emulsification and favorable changes on the cell surface, allowing it to enter *Rhodococcus* sp. D1 cells, and degradation subsequently occurred [54]. The glycolipid produced from the *Rhodococcus* sp. strain IITRO3 also makes the greater impact on degradation of 1,1,1-trichloro-2,2-bis (4-chlorophenyl) ethane [55]. The distribution of glycoconjugate-producing bacteria was reported in contaminated arid southwestern soil [56]. Rhizospheric microbes play an important role in the degradation of soil contamination, enhancing the degradation found with production of the glycoconjugates [57].

Another important concern is the effect of glycoconjugate biosurfactants on the candidate microbial strains that degrade OPs. The contrasting strains of *P. aeruginosa* produce glycoconjugate biosurfactants that enhance solubility and metabolism [58]. The purified biosurfactants cause an increase in the solubility of pyrene and higher solubilization of fluorene. The concentration of the biosurfactants is also very important for microbial growth. A higher concentration of these glycoconjugates inhibits the growth of microbial cells and reduces biodegradation potential [59]. These reports are not same for all the microbial strains, however, sometimes, a low concentration of glycoconjugate biosurfactants might also be toxic and show an antimicrobial effect [60, 61]. Most biodegradation of OPs has been reported previously with axenic microbial strains, whereas for the consortium, more biodegradation was achieved. The glycoconjugates increased the rate of OP degradation with a microbial consortium due to cumulative effect of microbial communities [62]. In a study a seawater *B. methylotrophicus* produced glycoconjugate biosurfactants that reduce surface tension, can be used for bioremediation purposes [63]. Microbial rhamnolipids and surfactin are used by researchers for the bioremediation of organic pollutants in last decades [64, 65]. The advantage of biosurfactants over synthetic surfactants is that the former induces low toxicity and stability in the presence of high temperature, high pH, and saline environment [66]. Natural glycoconjugate surfactants play a role in sustainable development and bioremediation [67].

Substrates containing the rich carbohydrates and lipids have been recommended for large-scale microbial glycoconjugate production [65]. The most commonly used substrates viz., corn liquor, glycerol, soybean oil, animal fat, vegetable fat, and molasses [68–72]. The previous study concluded that agro-industrial waste can also be used for microbial glycoconjugate production [73]. These carbohydrate- and lipid-containing compounds are metabolized by microbial metabolic pathways and converted into glycoconjugates such as rhamnolipid. The choice of substrate for microbial growth determines the amount of glycoconjugate production. Microbes are able to produce glycoconjugates from all types of carbon sources, but to achieve higher production, soybeans, corn, canola, and olives can be used (Table 2). Glycoconjugates are considered secondary metabolites due to their production in the stationary phase of microbial growth [37].

**Table 2:** Glycoconjugates producing microorganisms and the associated techniques

Microorganisms	Nature of glycoconjugates	Types of glycoconjugates	Techniques used for identification	References
*Pseudomonas aeruginosa* MA01	Glycolipid	Monorhamnolipid, dirhamnolipid	Fourier transform infrared spectroscopy (FTIR), thin layer chromatography (TLC)	[74]
*Acinetobacter baumannii*	Glycolipid	Palmitic and phthalic acid	FTIR, gas chromatography and masss pectrometry (GC-MS), nuclear magnetic resonance (NMR)	[17]
*Pseudomonas aeruginosa* PG1	Glycolipid	Mono and di rhamnolipid congeners	FTIR, liquid chromatography-mass spectrometry (LC-MS), and scanning electron microscope-energy dispersive spectrometer (SEM-EDS)	[9]
*Pseudomonas* sp.	Glycolipid	Rhamnolipid	FTIR spectra analysis	[75]
*Pseudoxanthomonas* sp. G3	Glycolipid	Rhamnolipid type	FTIR spectra analysis	[76]
*Lactobacillus casei*	Glycoprotein	Glycoprotein	FTIR and NuPAGE method	[77]
*Vibrio* sp. 3B-2	Glycoprotein	Glycoprotein	Chemical method, spectrometric characterization	[78]
*Candida bombicola* ATCC 22,214	Glycolipid	Sophorolipid	NMR, high performance thin layer chromatography (HPTLC) and MALDI ToF MS	[79]
*Starmerella bombicola*	Glycolipid	Sophorolipid	FTIR	[80]
*Rhodococcus* sp. PML026	Glycolipid	Trehalolipids	LC-MS	[81]
*Rhodococcus* sp. PML026	Glycolipid	Trehalolipids	Chemical analysis	[82]
*Cryptococcus Humicola* JCM 1461	Glycolipid	Cellobiose lipid	Chemical analysis	[83]
*Streptomyces* sp. DPUA 1559	Glycoprotein	Low mol. wt. glycoprotein	Electrophoretic analysis	[84]
*Ochrobactrum anthropi* HM-1	Glycolipid	Rhamnolipid type	TLC and FTIR spectra analysis	[85]
*Citrobacter freundii* HM-2	Glycolipid	Rhamnolipid type	TLC and FTIR spectra analysis	[85]
*Lactobacillus*	Glycoprotein	--	TLC and FTIR	[86]
*Pseudomonas* isolate DYNA270	Glycolipid	Rhamnolipids	Mass spectrometry	[87]
*Streptomyces nocardiopsis* A17, *Bacillus subtilis* ICA56	Glycerol	Biosurfactant	TLC and LC-MS	[88]
*Bacillus psudomycoides* BS6	Lipopeptide	Fatty acid 3-OH and peptide of five amino acid	TLC and FTIR	[89]
*Bacillus subtilis* B20, *B. subtilis* B30	Glycolipopeptide	Surfactin	Pedant drop method, Lyophilization	[29]
*Pseudomonas aeruginosa*	Glycolipid	Rhamnolipid		[90]
*P. aeruginosa* MA01	Glycolipid	Monorhamnolipid	FTIR, electrospray ionization mass spectrometry (ESI-MS)	[74]
*Klebsiella pneumonae* WME02	Phospholipid	biosurfactant	Biochemical characterization	[91]
*Pseudomonas aeruginosa* DS10-129	Glycolipid	Rhamnolipid	Mass spectrometry	[92]
*Candia lipolytica* IA 1055	Glycolipid	Sophorolipid	Emulsification, spectrophotometer	[93]
*Pseudomonas aeruginosa*	Glycolipid	Rhamnolipid	Spectrophotometer	[94]
*Serratia marcescens* UCP 1549	Glycolipid	Biosurfactant	Emulsification	[95]
*Bacillus subtilis*	Glycolipopeptide	Cyclic lipopeptide biosurfactant	High performance liquid chromatography (HPLC), emulsification	[96, 97]
*Candia lipolytica* UCP0988	Glycolipid	Sophorolipids	TLC, HPLC-ESI-MS	[90]
*Marinobacter hydrocarbonoclasticus* SdK644	Glycolipid	Biosurfactant	GC-MS, FTIR	[98]
*Paenibacillus* sp. D9	Glycolipopeptide	Biosurfactant	Emulsification	[31, 97]
*Pseudozyma aphidis* ZJUDM34	Glycolipid	Mannosylerythritol lipids	TLC, GC-MS	[99]
*Bacillus subtilis*, *Paenibacillus* sp. D9	Glycolipopeptide	Surface active biosurfactant	Gene cloning and expression, affinity chromatography	[30, 97]

### Glycoconjugates in action: overview of biofilm formation

In recent years, researchers have taken great interest in the biofilm-based degradation of environmental contaminants. Microbial glycoconjugates also play an important role in biofilm formation and accelerate the bioremediation of the organic pollutants. Generally, under laboratory conditions, a single microbial strain is isolated to test its biodegradation potential for environmental contaminants. However, the basic facts of the environmental interactions between the chosen microbial cell and other microbial communities, or the nature of their habitats, are ignored [100, 101]. Therefore, to harness the potential of microbial cells for glycoconjugate production and impact on biodegradation, it is necessary to consider the behavior of microbial communities and their habitats, even though the experiment was performed under laboratory conditions [5]. In nature, microbes interact with abiotic and biotic factors and produced the glycoconjugates. To maintain their populations via different types of interactions such as synergistic and antagonistic effects that allow microbes to adapt to different environmental conditions at polluted sites. Microbial communities consist of various microbial species that produced the glycoconjugate surfactants which have greater potential than a single culture glycoconjugates because the number of reporting genes and the diversity of metabolic activities work together and provide the maximum output within the shortest period [102]. So, the glycoconjugates produced by various microbial communities showed the cumulative effect on the degradation of the OPs. Importantly, the many microorganisms and microbial species present in microbial “biofilm” can degrade the wide range of contaminants present in the natural environment and engineered systems. Biofilm refers to a group of diverse microbial species attached to any living or nonliving surface and covered by a surrounding self-synthesized glycoconjugates, matrix comprising extracellular DNA, proteins, and water [103, 104]. Biofilm aids in the consumption of nutrients and oxygen, with tolerance against harsh environmental conditions during the bioremediation process. Biofilm based remediation technology is more cost-effective, ecofriendly, and easy for removing pollutants from the natural environment. Due to the production of glycoconjugates microbial biofilm absorbs and immobilizes environmental pollution, and the labor of gene expression divided among the existing microbial communities ultimately works very efficiently as a single unit. The various microbial communities within the biofilm are also responsible for differential gene expression of the substrate, showing a broad range of metabolic pathways for biodegradation. The most important characteristics of biofilm are their chemotaxis and flagellar-based movement. Biofilm can sense the presence of xenobiotics in their proximity and move towards them by swimming, swarming, and twitching, as well as by quorum sensing, which improves biodegradation in presence of glycoconjugates [105, 106]. The composition of microbial biofilm depends on the environmental conditions in which the microbes reside [107–109]. Biofilm provides better environmental conditions and protection from environmental stress, acid stress, antimicrobial stress, UV stress, desiccation, predation, biocides, solvent, toxic chemicals, and other pollutants [110, 111]. Microbial biofilms are increasingly used as indicator systems for monitoring heavy metal contamination in water resources. Changes in the morphology of biofilms and their physiology indicate the occurrence of contaminants in their proximity. Biofilm is frequently found in different geographical locations, such as streambeds, tidal flats, corroded pipes, and sites of infection [112–114].

**Fig. 3:**
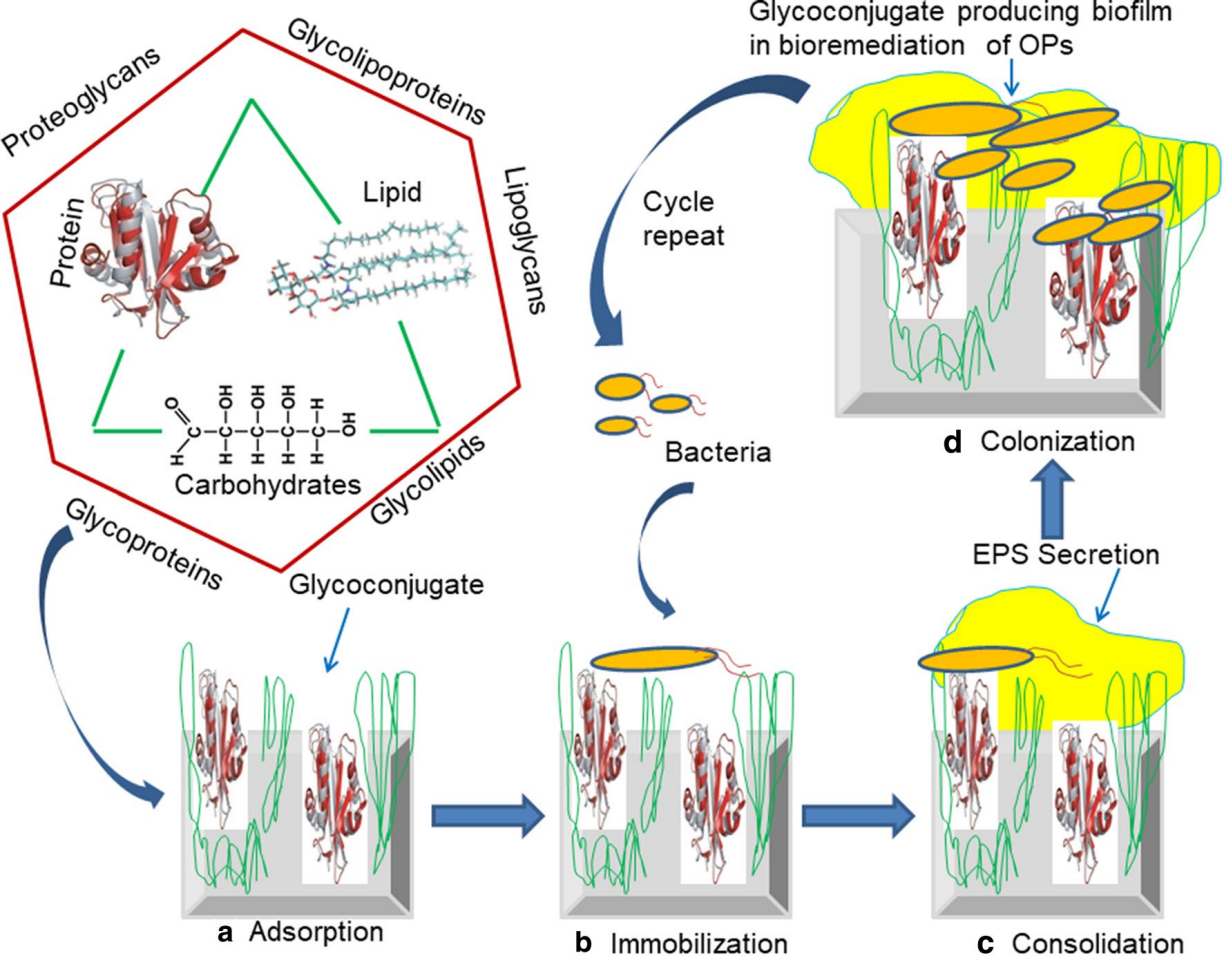
Role of glycoconjugate in biofilm formation included different steps. Carbohydrate, lipids and protein unite together and form glycoconjugate; **a** adsorption: attachment of carbohydrates and proteins to the surface of substrate; **b** immobilization of microbial cells on the surface of glycoconjugate. **c** consolidation: secretion of extracellular polymeric substance (EPS) by immobilized microbial cells on the cell surface; **d** colonization: microbial cells replicate and secreted large amount of glycoconjugates and forms biofilm which play role in bioremediation of OPs

Microbes are able to communicate with each other in the form of communities and biofilms. The main mechanism behind biofilm formation is quorum sensing. In addition to playing various other roles, glycoconjugates help microbial cells to attach to one another in a biofilm [27]. Glycoconjugates create a favorable environment for the biodegradation of the OPs at the contaminated sites. Microbial cells produce an extracellular matrix that helps the cells attach to each other in communities. Glycoconjugates also help the microbes survive under extreme conditions and protect the microbial cells from the outer environment, especially under water stress conditions. The adhesion of the bacterial cells occurs in both the mobile and stagnant phases. These glycoconjugates are useful for floating the bacterial cells in water bodies as a biofilm, which can be efficiently utilized for bioremediation in water systems. The critical factor of biofilm formation is the production of the glycoconjugate biosurfactant, smoothness at the cell surface, the velocity of flow, and bacterial growth [115]. Biofilm formation is governed by several signaling molecules and glycoconjugates. Naturally, biofilm formation is a complex process that involves many steps. Preliminary bacterial cells produce extracellular polymeric substances (EPSs). These substances act as cementing material on the outer cell membrane and help in the entrapment of nutrients. In addition, EPS also has surfactant properties that help in the mineralization of xenobiotic compounds that are otherwise inaccessible. The production of EPS and water form a slimy layer in biofilms. Microbes also engage in symbiotic relationships with each other at the polluted sites (Fig. 3). The intermediate metabolites produced by primary bacterial colonizers can be used by the secondary colonizers that ultimately form the biofilm. The quorum sensing (QS) mechanisms are well-established for these biofilms and help in regulating EPS production [116]. The QS system can help microbes survive in the presence of stress, such as antimicrobial compounds, nutrient limiting conditions, and OPs. Microbial strains degrade toxic chemicals through the establishment of cellular communications with each other [117].

Glycoconjugates also play an important role in aggregation of the microbial cells in communities. The aggregation of microbial cells is an essential factor in biofilm formation [118, 119]. Bacterial cells from two types of aggregation: auto and coaggregation. In auto-aggregation, genetically identical bacterial cells remain together, whereas coaggregation refers to genetically different cells [120]. The surface factors, extracellular polymeric substances, and diffusible signal molecules are critical factors involved in the auto-aggregation and microbial biofilm at polluted sites [121]. Aggregation also depends on microbial interactions such as antagonism, synergism, mutualism, competition, and commensalism [122].

Several *in-vitro* and *in-situ* studies based on biofilm have been conducted in recent decades in the field of bioremediation with glycoconjugates. *In-situ* biofilm mediated bioremediation can be performed in several ways. In nature, certain contaminants are degraded, transferred, and immobilized under specific environmental conditions without any interference of human activity [123]. Naturally, the biodegradation process requires the availability of the microorganism in the form of biofilm at polluted site and requires a long period of time. The addition of extra nutrients such as carbon, hydrogen, nitrogen, phosphorous, and oxygen to increase the growth rate of the microbial population enhances the degradation rate of pollutants [123, 124].

Biofilm can be used for the treatment of nitrates in wastewater [125] and biodegradation of the organic matter present in nature [126]. This biodegradation effort can be accelerated by designing a barrier material according to the concentration of the contaminant and the composition of the contaminant (mixed contaminant). The biodegradation process can be stimulated by providing added nutrients, electron acceptors and donors, or by providing a biocatalyst [51], which results in the development of biofilm on the surface of the contaminant via the natively present microbial species. If the existence of a required microorganism is lacking at the site of a contaminant, then the contaminant can be placed at a site where biofilm already exists. Alternatively, biofilm can be useful for the remediation of the toxic chemicals. Ultimately, a less harmful product can be formed by microbial biotransformation in nature due to the production of glycoconjugates without engineering the microorganism [113, 127].

**Fig. 4:**
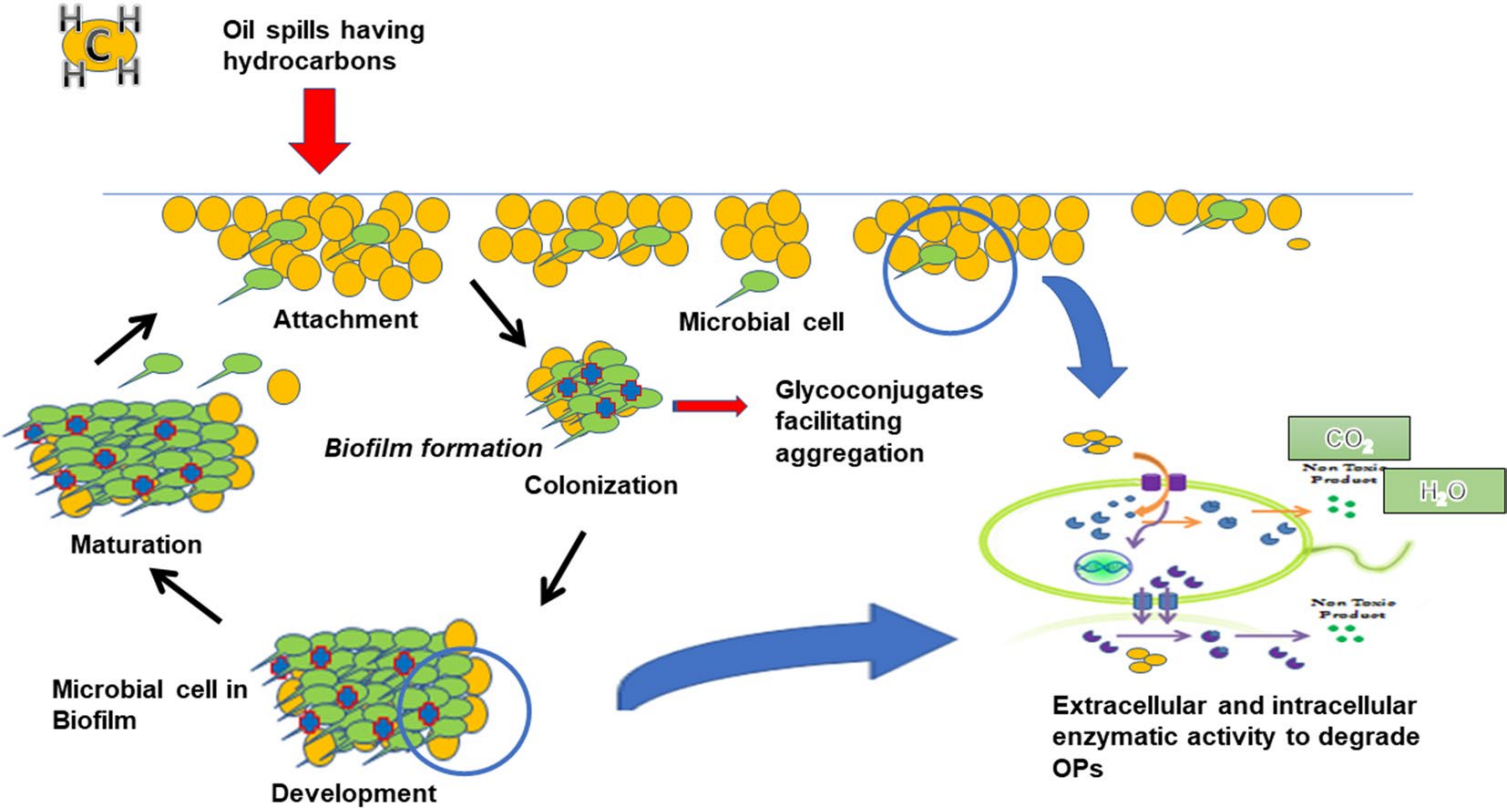
Mechanism of oil spill hydrocarbon degradation using microbial glycoconjugates

Generally, the *ex-situ* bioremediation process is performed in a bioreactor due to the unavailability of suitable microorganisms and the unfavorable conditions at a contaminated site. In bioreactors, biofilms are added as inert support and used for the biochemical conversion of pollutants by sorption, particularly heavy metals, hydrocarbons, industrial waste, and wastewater treatment [128–130]. Biofilm-based bioreactors have many advantages over conventional treatment methods. For example, a high concentration of pollutants can be treated for a longer period of time, the volumetric capacity of biodegradation is enhanced, and the tolerance for highly toxic xenobiotics is increased, thereby supporting anaerobic and aerobic metabolism together and reducing environmental interference. Industrial biofilm reactors are generally set up under special conditions, such as when freely floating microorganisms are unable to produce adequate biomass or the microbial biomass cannot be retained for a long enough time to convert the toxic pollutants to environmentally acceptable forms [130] (Fig. 4).

Bacterial and fungal biofilm is a special type of biofilm where the bacterial cell is attached to fungal hyphae. Fungal hyphae provide nutrients, increase the bioavailability to the bacterial cell, and enhance the rate of consumption of nutrients via competition. This enables the bacteria to search for nutrient by travelling through the fungal hyphae. Phenanthrene, a polyaromatic hydrocarbon of fused benzene rings, is associated with soil contamination. This compound is degraded by *Pseudomonas putida* PpG7 in the presence of *Pythium ultimum* fungal mycelia [131]. The previous researcher confirmed the importance of microbial glycoconjugates in biofilm formation and degradation of the OPs.

## Glycoconjugates in pesticide degradation

**Fig. 5:**
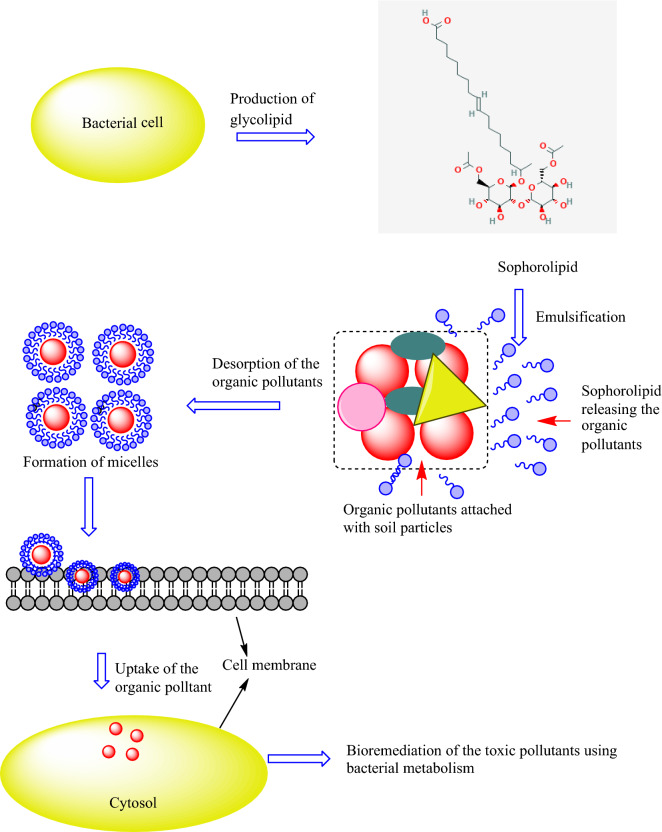
Microbial glycoconjugates facilitate organic pollutant degradation via micelle formation

Pesticides are organic compounds used in an enormous quantity in agriculture and homes to control a broad spectrum of pests [132, 133]. Most pesticides are hydrophobic with complex structures. Due to the large amounts of pesticides entering into soil and water systems, these molecules become attached to soil particles and are not available for microbial activities [134–139]. The attachment of pesticides to soil particles is dependent on the physical and chemical properties of the soil and pesticides [140]. Presently, various categories of pesticides are being sold in the market, such as organophosphates, organochlorines, and pyrethroids. The biodegradation of these pesticides is an intricate process due to their low water solubility and poor bioavailability. Microbial glycoconjugates play an important role in the desorption of pesticide molecules from soil particles. These glycoconjugate biosurfactant molecules decrease surface tension and enhance the degradation via microbial metabolism [33, 141]. Such types of microbial glycoconjugates are surface-active amphipathic emulsifying molecules that have the capacity to enhance the partitioning of hydrophobic pesticides to the aqueous phase by producing emulsions at and above their critical micellar concentration (CMC) (Fig. 5). This enhances the bioavailability of pesticides to their potential degraders and can thus play a crucial role in overcoming the above problems [142].

Microorganisms in the soil produce several types of glycoconjugates that induce emulsification of the contaminant and increase water solubility. The water solubility of pesticides is linked to their bioavailable fractions. The bioavailable fraction is used by microbial cells during metabolic activity [143, 144]. The glycoconjugate enhances pesticide degradation by reducing surface tension, modifying hydrophobicity, and enhancing bioavailability [145]. The glycoconjugates are reported to increase the solubility of the pesticides in soil and promote their degradation [146]. Due to the beneficial properties of the glycoconjugate, they are acceptable for use in contaminated sites [147]. Rhamnolipids, fructose lipids, sophorolipids, and glycolipopeptides are commonly investigated for pesticide bioremediation. In the last decade glycoconjugates, have emerged as a facilitator of pesticide degradation under various conditions [46, 148, 149]. We outline the major findings of glycoconjugates in the bioremediation of pesticides in Table 3.

**Table 3:** Biosurfactant mediated bioremediation of soils contaminated with pesticides

Pesticides	Concentration of pesticide	Biosurfactant/biosurfactant producing microbes	Degradation (%)	References
Organochlorines				
Dichlorodiphenyl trichloroethane (DDT)	282 µM	Trehalolipid from *Rhodococcus* sp. IITR03	50–60	[55]
DDT	1.417 mg/L	*Pseudomonas* sp. SB + *Grass* sp.	65.6	[150]
DDT	0.0474 mg/L	Rhamnolipids from *Arthrobacter globiformis*	64.3	[150]
DDT	0.25 µM	White rot fungi + biosurfactant from *Pseudomonas aeruginosa* and *Bacillus subtilis*	≈ 86	[151]
*α*-Endosulfan	200 mg/L	*Bacillus subtilis* MTCC 1427	100	[21]
Endosulfan soil	320 mg/L	*Pseudomonas aeruginosa* + rhamnolipid	> 90	[149]
*α*- and *β*-endosulfan	50 mg/L	*Arthrobacter* sp. ES-47	76.3–81.8	[152]
*α*- and *β*-endosulfan	50 mg/L	*Bordetella petrii* I GV 34 & GV36	82–89	[153]
*α*-Endosulfan	1420–3400 mg/L	Consortium of *Bordetella petrii* I GV 34 and *Bordetella petrii* II GV 36	100	[153]
*β*-Endosulfan	1280–3100 mg/L	*Achromobacter xylosoxidans* GV 47	100	[153]
Endosulfan	0.92 mg/L	Glycolipid, from *Pseudomonas* sp. B0406	Increased solubility	[154]
Endosulfan and hexachlorocyclohexane (HCH)	50 mg/L (endosulfan), 100 mg/L (HCH)	Rhamnolipid from *Lysinibacillus sphaericus* IITR51	Increased solubility	[155]
HCH	40 mg/L	Rhamnolipid from *Pseudomonas aeruginosa* + *Sphingomonas* sp. NM05	95	[156]
HCH	65 mg/L	*Cytisus striatus* plantation + *Rhodococcus erythropolis* ET54b	33	[157]
HCH	700 mg/L	*Pseudozyma* VITJzN01	3-9-fold increase in solubilization	[158]
HCH	–	Rhamnolipids	Increase solubility	[159]
Organophosphates				
Methyl Parathion	–	Glycolipid from *Pseudomonas* sp. B0406	Increased solubility	[154]
Chlorpyrifos	10 mg/L	*Pseudomonas* sp. ChlD + biosurfactant	> 98	[20, 143]
Methyl parathion and ethyl parathion	500 mg/L	Glycolipid from *Burkholderia cenocepacia* BSP3	Increased solubility	[18]
Quinalphos	10,000 mg/L	Biosurfactant from *Pseudomonas aeruginosa*	94	[159]

The addition of a glycoconjugate, an increased (30 %) biodegradation of endosulfan isomers by *B. subtilis* MTCC 1427 in both soil and liquids was reported in a previous study [23]. The enhanced mobilization and bioavailability of endosulfan isomers in the presence of the glycoconjugate was also reported and may be attributed to the enhanced solubilization of pesticides or the increased affinity towards microbial cells. The soil spiked with endosulfan showed enhanced degradation after 7 days of the experiment due to the production of rhamnolipids by *P. aeruginosa* [49]. A crude extract of a glycoconjugate (an anionic glycolipid) was produced by the *Pseudomonas* sp. B0406 strain and aided in the solubilization of endosulfan [154]. The *Lysinibacillus sphaericus* strain IITR51 was investigated as a way to produce a thermostable rhamnolipid glycoconjugate with the ability to enhance the solubility of the highly hydrophobic pesticide hexachlorocyclohexane (HCH) and endosulfan [155]. Bioaugmentation with the glycoconjugate-producing bacteria also proved to be an efficient technique for the remediation of pesticides. The *α* and *β* isomers of the endosulfan degraded by up to 82 % in the presence of glycocnjugates having biosurfactant properties [151, 152].

The bioavailable fractions of the lindane are poor in the environment, which hinders degradation via microbial actions. Lindane contains six chlorine atoms, which make it more persistent. The *Pseudomonas* Ptm^+^ strain was found to be a potent culture for the degradation of lindane in the environment along with the production of glycoconjugate. The production of the glycoconjugate was monitored in a minimal salt medium during lindane degradation. The produced glycoconjugate emulsified the organochlorine lindane to a greater extent than the other OPs [160]. A 95 % biodegradation rate was reported for lindane isomers by *Sphingomonas* sp. NM05 after the addition of rhamnolipids produced by *Pseudomonas aeruginosa* [156]. The impact of biosurfactants such as rhamnolipid, sophorolipid, and trehalose-containing lipid on the solubilization and biodegradation of HCH, and their isomers in soil were also studied [156]. It was observed that sophorolipids promote a higher degradation of HCH. The increased biodegradation of lindane (700 mg/L) by *Pseudozyma* VITJzN01 was demonstrated by a 3–9-fold increase in the solubilization of lindane isomers and was investigated with the addition of mannosylerythritol lipid bio-microemulsion [158]. Increased solubilization of lindane from 5 to 28 mg L^− 1^ was reported under an increasing concentration of rhamnolipids from 0 to 1000 mg L^− 1^ [144]. The trehalolipid produced by the *Rhodococcus* sp. strain IITR03 was isolated and characterized from the pesticide-contaminated sites [55]. Similarly, the effect of the rhamnolipid produced by *Arthrobacter globiformis* was investigated in the bioremediation of dichlorodiphenyl trichloroethane (DDT) [150]. Rhamnolipid enhanced the DDT degradation rate from 52 to 64 %. *Pseudomonas* sp. SB was able to produce a biosurfactant that promotes DDT degradation in combination with plant-microbe interactions [152]. The synergistic effects of mixed cultures of the white-rot fungus, *Pleurotus ostreatus*, and the biosurfactant-producing bacteria *Pseudomonas aeruginosa* and *Bacillus subtilis* on DDT biodegradation were investigated and found to enhance DDT degradation [151]. There are many ways to remediate contaminated soil with microbial treatments and other methods. Some of the most commonly applied methods include soil washing, vapor extraction, desorption, microbial consortium, and phytoremediation. Sodium dodecyl sulfate (SDS) and ethylene diamine tetra acetic acid (EDTA) were used to wash the soil with conventional methods. The combination of microbially produced citric acid and rhamnolipids is effective for the remediation of organochlorine pesticides from the soil [144]. Such microbial combinations are environmentally friendly and cost-effective and can help achieve environmental sustainability [160, 161].

The formation of stable emulsions was investigated using glycoconjugate produced by *Bacillus* strains and fenthion [26]. An anionic glycolipid produced by the *Pseudomonas* sp. B0406 strain was reported to aid in the solubilization of methyl parathion [154]. The complete degradation of chlorpyrifos (10 mg/L) was reported within 2 days of using *Pseudomonas* sp. supplemented with a glycoconjugate [21]. A > 10 times increase in the aqueous-phase solubility of chlorpyrifos was reported with the addition of a biosurfactant produced by *Pseudomonas* sp. [143]. The glycolipid from *Burkholderia cenocepacia* BSP3 isolated from oil-contaminated soil was proposed to possess the ability to bioremediate the pesticides methyl parathion and ethyl parathion [18]. It was observed that *Pseudomonas aeruginosa* produces a glycoconjugate that enhances the solubilization of quinalphos [159].

The glycoconjugate from *Pseudomonas cepacia* aided in degrading the hydrophobic herbicide 2,4,5-trichlorophenoxyaceticacid [162]. Similarly, a higher biodegradation of carbendazim was reported by adding rhamnolipid to *Rhodococcus* sp. D-1 [54]. Approximately 24–35 % biodegradation of trifluralin in the soil was reported after the addition of rhamnolipid [163]. The surfactin lipopeptide was produced by marine *Bacillus velezensis* MHNK1 under atrazine biodegradation. The complete degradation of atrazine was observed within 4 days after employing a combination of *B. velezensis* MHNK1 (2 %) and surfactin (2 CMC) [164].

### Glycoconjugates in wastewater treatment

Microbial glycoconjugates have emerged as a tool to clean wastewater contaminated with organic pollutants. Various microbial approaches are used for the bioremediation of wastewater, but glycoconjugates are gaining more attention. The activated sludge process is popular for wastewater treatment. This process is based on the aerobic digestion of the microbial strains that produce flocs (floc-forming microbes) [165]. These flocs are formed by the network of extracellular polymeric substances (EPSs) produced by microbes [166]. Bacterial strains have been reported for glycoconjugate production which consists of carbohydrates, proteins, humic substances, uronic acids, lipid compounds, and nucleic acids. Enzymes play an important role in the hydrolysis of sludge [167]. These enzymes help to release EPSs and identify polysaccharides and glycoconjugates together with a lectin panel [165, 168].

Effective glycoconjugates can reduce the surface tension of water from 72 to 25 mN/m and the interfacial tension between polar and non-polar liquids for water against n-hexadecane from 40 to 1 mN/m [169, 170]. Thus, glycoconjugates can also be used for the treatment of wastewater [171]. In a previous report, the enhanced removal of hydrocarbons was described using rhamnolipids, which was mainly attributed to improved solubility and reduced interfacial tension [172]. Microbial rhamnolipids are also described as efficient candidates for the pretreatment of waste activated sludge and contribute to the process of wastewater treatment [173]. *Rhodococcus* sp. PML026, a marine bacterial strain, was utilized for the production of glycoconjugate characterized as trehalolipids, exerted biosurfactant activity under diverse experimental conditions, and was proven to be an efficient candidate for wastewater treatment and other bioremediation purposes [174]. The various bacterial isolates for glycoconjugates were investigated by their biosurfactant producing abilities. These isolates have the potential to reduce the surface tension in the liquid medium from 71.1 mN/m to 32.1 mN/m. The isolates were mainly belonging to the Aeromonadaceae, Bacillaceae, Enterobacteriaceae, Gordoniaceae, and Pseudomonadaceae families [175]. The wastewater bacterial strains showed antibiotic resistance and biofilm formation due to the production of biosurfactants. Low surface tension values of 28 and 36 mN/m were observed in the bacteria, which were not able to form a biofilm. This study showed that low surface tension can produce a weak biofilm, which can be correlated to the glycoconjugate playing a role in effective biofilm formation at polluted sites [122, 176]. Hollow membranous fibers also developed. These fibers supply the dissolved hydrogen to microbial population that stimulate the biodegradation of the chlorinated solvent present in groundwater [123]. Sophorolipids are another glycoconjugate biosurfactant utilized in oil spill management and the oil biodegradation of contaminated water [124]. Thus, microbial glycoconjugates are utilized in diverse forms for the treatment of wastewater, and the results obtained justify their candidacy for this purpose [97, 177, 178].

## Conclusions and future prospects

Microbial glycoconjugates are important for bioremediation purposes, and several investigations have confirmed the degradation-specific role of glycoconjugates. The glycoconjugates can be used for the broad bioremediation of pesticides, hydrocarbons, antibiotics, and several xenobiotics. Microbial glycoconjugates play a key role in the adhesion of cells in biofilms that increase the degradation efficiency for OPs. Thus, recent advances in the field of MG have added to the potential of glycoconjugates in different applications along with the management of OPs, which are an environmental nuisance due to their intense utilization in different anthropogenic activities. MG bears many unexplored horizons to be revealed and utilized in the development of efficient bioremediation procedures. Recent high-throughput omics-based techniques could be applied to explore the molecular basis of the glycoconjugate-based bioremediation of OPs.

## Data Availability

Not applicable.

## References

[1] Bhatt P, Gangola S, Bhandari G, Zhang W, Maithani D, Mishra S, Chen S (2021). New insights into the degradation of synthetic pollutants in contaminated environments. Chemosphere.

[2] Chen T, Chang S (2020). Potential microbial indicators for better bioremediation of an aquifer contaminated with vinyl chloride or 1,1-dichloroethene. Water Air Soil Pollut.

[3] Bhatt P, Huang Y, Zhan H, Chen S (2019). Insight into microbial applications for the biodegradation of pyrethroid insecticides. Front Microbiol.

[4] Yoshikawa M, Zhang M, Toyota K (2017). Biodegradation of volatile organic compounds and their effects on biodegradability under co-existing conditions. Microbes Environ.

[5] Mishra S, Lin Z, Pang S, Zhang W, Bhatt P, Chen S (2021). Recent advanced technologies for the characterization of xenobiotic-degrading microorganisms and microbial communities. Front Bioeng Biotechnol.

[6] Yair S, Ofer B, Arik E, Shai S, Yossi R, Tzvika D, Amir K (2008). Organophosphate degrading microorganisms and enzymes as biocatalysts in environmental and personal decontamination applications. Crit Rev Biotechnol.

[7] Liston SD, Mann E, Whitfield C (2017). Glycolipid substrates for ABC transporters required for the assembly of bacterial cell-envelope and cell-surface glycoconjugates. Biochim Biophys Acta Mol Cell Biol Lipids.

[8] Chong H, Li Q (2017). Microbial production of rhamnolipids: Opportunities, challenges and strategies. Microb Cell Fact.

[9] Patowary K, Patowary R, Kalita MC, Deka S (2017). Characterization of biosurfactant produced during degradation of hydrocarbons using crude oil as sole source of carbon. Front Microbiol.

[10] Sattin S, Berbadi A (2016). Glycoconjugates and glycomimetics as microbial anti-adhesives. Trend Biotechnol.

[11] Enaime G, Nettmann E, Berzio S, Bacaoui A, Yaacoubi A, Wichern M, Gehring T, Lubken M (2020). Performance and microbial analysis during long-term anaerobic digestion of olive mill wastewater in a packed‐bed biofilm reactor. J Chem Technol Biotech.

[12] Messner P, Schaffer C, Kosma P (2013). Bacterial cell envelope glycoconjugates. Adv Carbohyd Chem Biochem.

[13] Tan YN, Li Q (2018). Microbial production of rhamnolipids using sugars as carbon sources. Microb Cell Fact.

[14] Zhou H, Huang X, Liang Y, Li Y, Xie Q, Zhang C, You S (2020). Enhanced bioremediation of hydraulic fracturing flowback and produced water using an indigenous biosurfactant-producing bacteria *Acinetobacter* sp, Y2. Chem Eng J.

[15] Aguila-Torres P, Maldonado J, Gaete A, Figueroa J, González A, Miranda R, González-Stegmaier R, Martin C, González M (2020). Biochemical and genomic characterization of the cypermethrin-degrading and biosurfactant-producing bacterial strains isolated from marine sediments of the Chilean Northern Patagonia. Mar Drugs.

[16] Deng Z, Jiang Y, Chen K, Li J, Zheng C, Gao F, Liu X (2020). One biosurfactant producing bacteria *Achromobacter* sp. A-8 and its potential use in microbial enhanced oil recovery and bioremediation. Front Microbiol.

[17] Gupta B, Puri S, Thakur IS, Kaur J (2020). Enhanced pyrene degradation by a biosurfactant producing *Acinetobacter baumannii* BJ5: Growth kinetics, toxicity and substrate inhibition studies. Environ Technol Innova.

[18] Wattanaphon HT, Kerdsin A, Thammacharoen C, Sangvanich P, Vangnai AS (2008). A biosurfactant from *Burkholderia cenocepacia* BSP3 and its enhancement of pesticide solubilization. J Appl Microbiol.

[19] Sharma S, Singh P, Raj M, Chadha BS, Saini HS (2009). Aqueous phase partitioning of hexachlorocyclohexane (HCH) isomers by biosurfactant produced by *Pseudomonas aeruginosa* WH-2. J Hazard Mater.

[20] Singh PB, Sharma S, Saini HS, Chadha BS (2009). Biosurfactant production by *Pseudomonas* sp. and its role in aqueous phase partitioning and biodegradation of chlorpyrifos. Lett Appl Microbiol.

[21] Awasthi N, Kumar A, Makkar R, Cameotra SS (1999). Biodegradation of soil-applied endosulfan in the presence of a biosurfactant. J Environ Sci Heal B.

[22] Onbasli D, Aslim B (2009). Effects of some organic pollutants on the exopolysaccharides (EPSs) produced by some *Pseudomonas* spp. strains. J Hazard Mater.

[23] Zhou MF, Yuan XZ, Zhong H, Liu ZF, Li H, Jiang LL, Zeng GM (2011). Effect of biosurfactants on Laccase production and phenol biodegradation in solid-state fermentation. Appl Biochem Biotechnol.

[24] Zhang C, Wang S, Yan Y (2011). Isomerization and biodegradation of beta-cypermethrin by *Pseudomonas aeruginosa* CH7 with biosurfactant production. Bioresour Technol.

[25] Mnif I, Ghribi D (2016). Glycolipid biosurfactants: main properties and potential applications in agriculture and food industry. J Sci Food Agric.

[26] Patel S, Homaei A, Patil S, Daverey A (2018). Microbial biosurfactants for oil spill remediation: Pitfalls and potentials. Appl Microbiol Biotech.

[27] Karlapudi AP, Venkateswarulu TS, Tammineedi J, Kanumuri L, Ravuru BK, Drisala VR, Kodali VP (2018). Role of biosurfactants in bioremediation of oil pollution- a review. Petroleum.

[28] Casullo de Araújo HW, Fukushima K, Takaki GMC (2010). Prodigiosin production by *Serratia marcescens* UCP 1549 using renewable resources as a low-cost substrate. Molecules.

[29] Al-Bahry SN, Al-wahaibi YM, Elshafie AE, Al-Bemani AS, Joshi SJ, Al-Makhmari HS, Al-Sulaimani HS (2012). Biosurfactant production by *Bacillus subtilis* B20 using date molasses and its possible application in enhanced oil recovery. Int Biodeter Biodegr.

[30] Jimoh AA, Lin J (2018). Enhancement of *Paenibacillus* sp. D9 lipopeptide biosurfactant production through the optimization of medium composition and its application for biodegradation of hydrophobic pollutants. Appl Biochem Biotechnol.

[31] Jimoh AA, Lin J, Biosurfactant (2019). A new frontier for greener technology and environmental sustainability. Ecotoxicol Environ Saf.

[32] Lee DW, Lee H, Kwon BO, Khim JS, Yim UH, Kim BS, Kim JJ (2018). Biosurfactant assisted bioremediation of crude oil of by indigenous bacteria isolated from Taean beach sediment. Environ Pollut.

[33] Banat IM, Franzetti A, Gandolfi I, Bestetti G, Martinotti MG, Fracchia L, Smyth TJ, Marchant R (2010). Microbial biosurfactants production, applications and future potential. Appl Microbiol Biotechnol.

[34] Moya RI, Tsaousi K, Rudden M, Marchant R, Alameda EJ, Garcia RM, Banat IM (2015). Rhamnolipid and surfactin production from olive oil mill waste as sole carbon source. Bioresour Technol.

[35] Varjani SJ, Upasani VN (2016). Core flood study for enhanced oil recovery through ex-situ bioaugmentation with thermo- and halo-tolerant rhamnolipid produced by *Pseudomonas aeruginosa* NCIM 5514. Bioresour Technol.

[36] Varjani SJ, Rana DP, Jain AK, Bateja S, Upasani VN. Synergistic ex-situ biodegradation of crude oil by halotolerant bacterial consortium of indigenous strains isolated from on shore sites of Gujarat India. Bioresour Technol*.* 2015; 103: 116–124.

[37] Soberon-Chavez G, Lepine F, Deziel E (2005). Production of rhamnolipids *Pseudomonas aeruginosa*. Appl Microbiol Biotechnol.

[38] Varjani SJ, Upasani VN (2017). Critical review on biosurfactant analysis, purification and characterization using rhamnolipid as a model biosurfactant. Bioresour Technol.

[39] Jezierska S, Claus S, Van Bogaert I (2018). Yeast glycolipid biosurfactants. FEBS Lett.

[40] Sun M, Ye M, Wu J, Feng Y, Shen F, Tian D, Liu K, Hu F, Li H, Jiang X, Yang L, Kengara FO (2015). Impact of bioaccessible pyrene on the abundance of antibiotic resistance genes during *Sphingobium* sp.- and sophorolipid-enhanced bioremediation in soil. J Hazard Mater.

[41] Kosaric N (2001). Biosurfactants and their application for soil bioremediation. Food Technol Biotechnol.

[42] Lai CC, Huang YC, Wei YH, Chang JS (2009). Biosurfactant enhanced removal of total petroleum hydrocarbons from contaminated soil. J Hazard Mater.

[43] Inakollu S, Hung H, Shreve GS (2004). Biosurfactant enhancement of microbial degradation of various structural classes of hydrocarbon in mixed waste systems. Environ Eng Sci.

[44] Whang LM, Liu PWG, Ma CC, Cheng SS (2009). Application of rhamnolipid and surfactin for enhanced diesel biodegradation-effects of pH and ammonium addition. J Hazard Mater.

[45] Franzetti A, Caredda P, Ruggeri C, La Colla P, Tamburini E, Papacchini M, Bestetti G (2009). Potential applications of surface-active compounds by *Gordonia* sp. strain BS29 in soil remediation technologies. Chemosphere.

[46] Mata-Sandoval JC, Karns J, Torrents A (2001). Effect of nutritional and environmental conditions on the production and composition of rhamnolipids by *P. aeruginosa* UG2. Microbiol Res.

[47] Moldes AB, Paradelo R, Rubinos D, Devesa-Rey R, Cruz JM, Barral MT (2011). *Ex situ* treatment of hydrocarbon-contaminated soil using biosurfactants from *Lactobacillus pentosus*. J Agric Food Chem.

[48] Sachdev DP, Cameotra SS (2013). Biosurfactants in agriculture. Appl Microbiol Biotechnol.

[49] Karanth NGK, Deo PG, Veenanadig NK (1999). Microbial productions of biosurfactants and their importance. Curr Sci.

[50] Chrzanowski Ł, Wick LY, Meulenkamp R, Kaestner M, Heipieper HJ (2009). Rhamnolipid biosurfactants decrease the toxicity of chlorinated phenols to *Pseudomonas putida* DOT-T1E. Lett Appl Microbiol.

[51] Chrzanowski T, Owsianiak M, Szulc A, Marecik R, Piotrowska-Cyplik A, Olejnik-Schmidt AK, Staniewski J, Lisiecki P, Ciesielczyk F, Jesionowski T, Heipieper HJ (2011). Interactions between rhamnolipid biosurfactants and toxic chlorinated phenols enhance biodegradation of a model hydrocarbon-rich effluent. Int Biodeterior Biodegrad.

[52] Sponza DT, Gok O (2011). Effects of sludge retention time and biosurfactant on the treatment of polyaromatic hydrocarbon (PAH) in a petrochemical industry wastewater. Water Sci Technol.

[53] Alvarez A, Saez JM, Costa JSD, Colin VL, Fuentes MS, Cuozzo SA, Benimeli CS, Polti MA, Amoroso MJ (2017). *Actinobacteria*: Current research and perspectives for bioremediation of pesticides and heavy metals. Chemosphere.

[54] Bai N, Wang S, Abuduaini R, Zhang M, Zhu X, Zhao Y. Rhamnolipid-aided biodegradation of carbendazim by *Rhodococcus* sp. D-1: Characteristics, products, and phytotoxicity. Sci Total Environ*.* 2017, 590: 343–351. 10.1016/j.scitotenv.2017.03.025 28279530

[55] Bajaj A, Mayilraj S, Mudiam MKR, Patel DK, Manickam N (2014). Isolation and functional analysis of a glycolipid producing *Rhodococcus* sp. strain IITR03 with potential for degradation of 1,1,1-trichloro-2,2-bis(4-chlorophenyl) ethane (DDT). Bioresour Technol.

[56] Bodour AA, Drees KP, Maier RM (2003). Distribution of biosurfactant producing bacteria in undisturbed and contaminated arid southwestern soils. Appl Environ Microbiol.

[57] dos Santos JJ, Moranho LT (2018). Rhizospheric microorganisms as a solution for the recovery of soils contaminated by petroleum: A review. J Environ Manage.

[58] Bordoloi NK, Konwar BK (2009). Bacterial biosurfactant in enhancing solubility and metabolism of petroleum hydrocarbons. J Hazard Mater.

[59] Whang LM, Liu PWG, Ma CC, Cheng SS (2008). Application of biosurfactants, rhamnolipid, and surfactin, for enhanced biodegradation of diesel-contaminated water and soil. J Hazard Mater.

[60] Vatsa P, Sanchez L, Clement C, Baillieul F, Dorey S (2010). Rhamnolipid biosurfactants as new players in animal and plant defense against microbes. Int J Mol Sci.

[61] Lima TMS, Procópio LC, Brandão FD, Carvalho AMX, Tótola MR, Borges AC (2011). Biodegradability of bacterial surfactants. Biodegradation.

[62] Lawnicjak L, Marecik R, Chrzanonowski L (2013). Contribution of biosurfactants to natural or induced bioremediation. Appl Microbiol Biotechnol.

[63] Chaprao MJ, da Silva RCFS, Rufino RD, Luna JM, Santos VA, Sarubbo LA (2018). Production of a biosurfactant from *Bacillus methylotrophicus* UCP1616 for use in the bioremediation of oil-contaminated environments. Ecotoxicology.

[64] Campos JM, Stamford TLM, Sarubbo LA, Luna JM, Rufino RD, Banat IM (2013). Microbial biosurfactants as additives for food industries. Biotechnol Prog.

[65] Santos DKF, Rufino RD, Luna JM, Santos VA, Sarubbo LA (2016). Biosurfactants: Multifunctional biomolecules of the 21st century. Int J Mol Sci.

[66] Almeida DG, Silva MGC, Barbosa RN, Silva DSP, Silva RO, Lima GMS, Gusmão NB, Sousa MFVQ (2017). Biodegradation of marine fuel MF-380 by microbial consortium isolated from seawater near the petrochemical Suape Port, Brazil. Int Biodeterior Biodegrad.

[67] Silva RCFS, Almeida DG, Rufino RD, Luna JM, Santos VA, Sarubbo LA (2014). Applications of biosurfactants in the petroleum industry and the remediation of oil spills. Int J Mol Sci.

[68] Rocha e Silva NMP, Rufino RD, Luna JM, Santos VA, Sarubbo LA (2014). Screening of *Pseudomonas* species for biosurfactant production using low-cost substrates. Biocatal Agric Biotechnol.

[69] Silva SNRL, Farias CBB, Rufino RD, Luna JM, Sarubbo LA (2010). Glycerol as substrate for the production of biosurfactant by *Pseudomonas aeruginosa* UCP0992. Colloids Surf B Biointerf.

[70] Luna JM, Santos Filho AS, Rufino RD, Sarubbo LA (2016). Production of biosurfactant from *Candida bombicola* URM 3718 for environmental applications. Chem Eng Trans.

[71] Gusmão CAB, Rufino RD, Sarubbo LA (2010). Laboratory production and characterization of a new biosurfactant from *Candida glabrata* UCP1002 cultivated in vegetable fat waste applied to the removal of hydrophobic contaminant. World J Microbiol Biotechnol.

[72] Almeida DG, Soares da Silva RCF, Luna JM, Rufino RD, Santos VA, Sarubbo LA (2017). Response surface methodology for optimizing the production of biosurfactant by *Candida tropicalis* on industrial waste substrates. Front Microbiol.

[73] Rivera AD, Urbina MAM, Lopez VEL (2019). Advances on research in the use of agro–industrial waste in biosurfactant production. World Microbiol Biotech.

[74] Abbasi H, Hamedi MM, Lotfabad TB, Zahiri HS, Sharafi H, Masoomi F, Moosavi-Movahedi AA, Ortiz A, Amanlou M, Noghabi KA (2012). Biosurfactant-producing bacterium, *Pseudomonas aeruginosa* MA01 isolated from spoiled apples: physicochemical and structural characteristics of isolated biosurfactant. J Biosci Bioeng.

[75] Mahalingam NU, Sampath N (2014). Isolation, characterization and identification of bacterial biosurfactant. Pela Res Lib.

[76] Astuti DI, Purwasena IA, Putri RE, Amaniyah M, Sugai Y (2019). Screening and characterization of biosurfactant produced by *Pseudoxanthomonas* sp. G3 and its applicability for enhanced oil recovery. J Petrol Explor Prod Technol.

[77] Goŀek P, Bednarski W, Brzozowski B, Dziuba B (2009). The obtaining and properties of biosurfactants synthesized by bacteria of the genus *Lactobacillus*. Ann Microbiol.

[78] Hu X, Wang C, Wang P (2015). Optimization and characterization of biosurfactant production from marine *Vibrio* sp. strain 3B-2. Front Microbiol.

[79] Elshafie AE, Joshi SJ, Al-Wahaibi YM, Al-Bemani AS, Al-Bahry SN, Al-Maqbali D, Banat IM (2015). Sophorolipids production by *Candida bombicola* ATCC 22214 and its potential application in microbial enhanced oil recovery. Front Microbiol.

[80] Liu Z, Tian X, Chen Y, Lin Y, Mohsin A, Chu J (2019). Efficient sophorolipids production via a novel *in situ* separation technology by *Starmerella bombicola*. Proc Biochem.

[81] Bages-Estopa S, White DA, Winterburn JB, Webb C, Martina PJ (2018). Production and separation of a trehalolipid biosurfactant. Biochem Engi.

[82] White DA, Hird LC, Ali ST (2013). Production and characterization of a trehalolipid biosurfactant produced by the novel marine bacterium *Rhodococcus* sp., strain PML026. J Appl Microbiol.

[83] Morita T, Ishibashi Y, Fukuoka T (2011). Production of glycolipid biosurfactants, cellobiose lipids, by *Cryptococcus humicola* JCM 1461 and their interfacial properties. Biosci Biotechnol Biochem.

[84] Santos A, Silva M, Costa E, Rufino RD, Santos VA, Ramos CS, Sarubbo LA, Porto A (2017). Production and characterization of a biosurfactant produced by *Streptomyces* sp. DPUA 1559 isolated from lichens of the Amazon region. Braz Med Biol Res.

[85] Ibrahim HMM (2018). Characterization of biosurfactants produced by novel strains of *Ochrobactrum anthropi* HM-1 and *Citrobacter freundii* HM-2 from used engine oil-contaminated soil. Egypt Petroliu.

[86] Ruangprachaya F, Chuenchomrat P (2018). Isolation and characterization of biosurfactant produced by Lactic acid bacteria from indigenous Thai fermented foods. Int J Food Engi.

[87] Shreve GS, Makula R (2019). Characterization of a new rhamnolipid biosurfactant complex from *Pseudomonas* Isolate DYNA270. Biomolecules.

[88] Chakraborty S, Ghosh M, Chakraborti S, Jana S, Sen KK, Kokare C, Zhang L (2015). Biosurfactant produced from *Actinomycetes nocardiopsis* A17: Characterization and its biological evaluation. Int J Biol Macromol.

[89] Li J, Deng M, Wang Y, Chen W (2016). Production and characteristics of biosurfactant produced by *Bacillus pseudomycoides* BS6 utilizing soybean oil waste. Int Biodeter Biodegrad.

[90] Santos DKF, Resende AHM, de Almeida DG, da Silva RCFS, Rufino RD, Luna JM, Banat IM, Sarubbo LA (2017). *Candida lipolytica* UCP0988 biosurfactant: Potential as a bioremediation agent and in formulating a commercial related product. Front Microbiol.

[91] Jamal P, Nawawi W, Alam MZ (2012). Optimum medium components for biosurfactant production by *Klebsiella pneumoniae* WMF02 utilizing sludge palm oil as a substrate. Aust J Basic Appl Sci.

[92] Rahman K, Rahman TJ, McClean S, Marchant R, Banat IM (2002). Rhamnolipid biosurfactant production by strains of *Pseudomonas aeruginosa* using low-cost raw materials. Biotechnol Prog.

[93] Vance-Harrop MH, Gusmao, NBd, Campos-Takak (2003). GMd.New bioemulsifiers produced by *Candida lipolytica* using D-glucose and babassu oil as carbon sources. Braz J Microbiol.

[94] Sobri IM, Halim M, Lai OM, Lajis AF, Yusof MT, Halmi MIE, Johari WLW, Wasoh H (2018). Emulsification characteristics of rhamnolipids by *Pseudomonas aeruginosa* using coconut oil as carbon source. J Environ Microbiol Toxicol.

[95] Araujo HWC, Andrade RFS, Montero-Rodriguez D, Rubio-Ribeaux D, Alves da Silva CA, Campos-Takaki GM (2019). Sustainable biosurfactant produced by *Serratia marcescens* UCP 1549 and its suitability for agricultural and marine bioremediation applications. Microb Cell Fact.

[96] Felix AKN, Martins JLL, Almeida JGL, Giro MEA, Cavalcante KF, Melo VMM, Pessoa ODL, Rocha MVP, Goncalves LRB, Aguiar RSS (2019). Purification and characterization of a biosurfactant produced by *Bacillus subtilis* in cashew apple juice and its application in the remediation of oil contaminated soil. Colloid Surface B.

[97] Saborimanesh N, Mulligan CN (2015). Effect of sophorolipid biosurfactant on oil biodegradation by the natural oil-degrading bacteria on the weathered biodiesel, diesel and light crude oil. J Bioremed Biodeg.

[98] Zenati B, Chebbi A, Badis A, Eddouaouda K, Boutoumi H, El Hattab M, Hentati D, Chelbi M, Sayadi S, Chamkha M, Franzetti A (2018). A non-toxic microbial surfactant from *Marinobacter hydrocarbonoclasticus* SdK644 for crude oil solubilization enhancement. Ecotoxicol Environ Saf.

[99] Niu Y, Wu J, Wang W, Chen Q (2019). Production and characterization of a new glycolipid, mannosylerythritol lipid, from waste cooking oil biotransformation by *Pseudozyma aphidis* ZJUDM34. Food Sci Nutr.

[100] Mishra S, Zhang W, Lin Z, Pang S, Huang Y, Bhatt P, Chen S (2020). Carbofuran toxicity and its microbial degradation in contaminated environments. Chemosphere.

[101] Huang Y, Zhang W, Pang S, Chen J, Bhatt P, Mishra S, Chen S (2021). Insights into the microbial degradation and catalytic mechanisms of chlorpyrifos. Environ Res.

[102] Gaur N, Flora G, Yadav M, Tiwari A (2014). A review with recent advancements on bioremediation-based abolition of heavy metals. Environ Sci-Proc Imp.

[103] Gieg LM, Fowler SJ, Berdugo-Clavijo C (2014). Syntrophic biodegradation of hydrocarbon contaminants. Curr Opin Biotech.

[104] Horemans B, Breugelmans P, Hofkens J, Smolders E, Springael D (2013). Environmental dissolved organic matter governs biofilm formation and subsequent linuron degradation activity of a linuron-degrading bacterial consortium. Appl Environ Microbiol.

[105] Pratt LA, Kolter R (1999). Genetic analyses of bacterial biofilm formation. Curr Opinion Microbiol.

[106] Lacal J, Reyes-Darias JA, García‐Fontana C, Ramos JL, Krell T (2013). Tactic responses to pollutants and their potential to increase biodegradation efficiency. J Appl Microbiol.

[107] Field JA, Stams AJ, Kato M, Schraa G (1995). Enhanced biodegradation of aromatic pollutants in cocultures of anaerobic and aerobic bacterial consortia. Anton Leeuw.

[108] Kreft JU, Wimpenny JW (2001). Effect of EPS on biofilm structure and function as revealed by an individual-based model of biofilm growth. Water Sci Technol.

[109] Miqueleto AP, Dolosic CC, Pozzi E, Foresti E, Zaiat M (2010). Influence of carbon sources and C/N ratio on EPS production in anaerobic sequencing batch biofilm reactors for wastewater treatment. Bioresour Technol.

[110] Mah TFC, O’Toole GA (2001). Mechanisms of biofilm resistance to antimicrobial agents. Trends Microbiol.

[111] Sutherland IW (2001). The biofilm matrix an immobilized but dynamic microbial environment. Trends Microbiol.

[112] Latch DE, Packer JL, Arnold WA, McNeill K (2003). Photochemical conversion of triclosan to 2, 8-dichlorodibenzo-p-dioxin in aqueous solution. J Photoch Photobio A.

[113] Seo Y, Lee WH, Sorial G, Bishop PL (2009). The application of a mulch biofilm barrier for surfactant enhanced polycyclic aromatic hydrocarbon bioremediation. Environ Pollut.

[114] Wang Y, Oyaizu H (2011). Enhanced remediation of dioxins-spiked soil by a plant–microbe system using a dibenzofuran-degrading *Comamonas* sp. and *Trifolium repens* L. Chemosphere.

[115] Donlan RM, Costerton JW (2002). Biofilms: survival mechanisms of clinically relevant microorganisms. Clinical Microbiol Rev.

[116] Fletcher M, Lessman JM, Loeb GI (1991). Bacterial surface adhesives and biofilm matrix polymers of marine and freshwater bacteria. Biofouling.

[117] Zhang W, Pang S, Lin Z, Mishra S, Bhatt P, Chen S (2021). Biotransformation of perfluoroalkyl acid precursors from various environmental systems: Advances and perspectives. Environ Pollut.

[118] Singh R, Paul D, Jain RK (2006). Biofilms: Implications in bioremediation. Trends Microbiol.

[119] Simoes LC, Simoes M, Vieira MJ (2007). Biofilms interactions between distinct bacterial genera isolated from drinking water. Appl Environ Microbiol.

[120] Katharios-Lanwermeyer S, Xi C, Jakubovics NS, Rickard AH (2014). Mini-review: microbial coaggregation: ubiquity and implications for biofilm development. Biofouling.

[121] Bogino PC, De las Mercedes OM, Sorroche FG, Giordano W (2013). The role of bacterial biofilms and surface components in plant-bacterial associations. Int J Mol Sci.

[122] Jaloweicki L, Zur J, Cojniak J, Ejhed H, Plaza G (2017). Properties of antibiotic-resistant bacteria isolated from onsite wastewater treatment plant in relation to biofilm formation. Curr Microbiol.

[123] Jorgensen KS, Salminen JM, Bjorklof K, cummings SP (2010). Monitored natural attenuation. Bioremediation: Methods and Protocols.

[124] Vogt C, Richnow HH (2013). Bioremediation via *in situ* microbial degradation of organic pollutants. Geobiotechnology II.

[125] Williamson WM, Close ME, Leonard MM, Webber JB, Lin S (2012). Groundwater biofilm dynamics grown in situ along a nutrient gradient. Groundwater.

[126] Långmark J, Storey MV, Ashbolt NJ, Stenström TA (2004). Artificial groundwater treatment: Biofilm activity and organic carbon removal performance. Water Res.

[127] Payne RB, May HD, Sowers KR (2011). Enhanced reductive dechlorination of polychlorinated biphenyl impacted sediment by bioaugmentation with a dehalorespiring bacterium. Environ Sci Technol.

[128] Bryers JD (1993). Bacterial biofilms. Curr Opinion Biotechnol.

[129] Boon N, De Gelder L, Lievens H, Siciliano SD, Top EM, Verstraete W (2002). Bioaugmenting bioreactors for the continuous removal of 3-chloroaniline by a slow release approach. Environ Sci Technol.

[130] Qureshi N, Annous BA, Ezeji TC, Karcher P, Maddox IS (2005). Biofilm reactors for industrial bioconversion processes: Employing potential of enhanced reaction rates. Microb Cell Fact.

[131] Day SM (1993). US environmental regulations and policies their impact on the commercial development of bioremediation. Trends Biotechnol.

[132] Bhatt P, Pal K, Bhandari G, Barh A (2019). Modeling of methyl halide biodegradation on bacteria and its effect on other environmental systems. Pest Biochem Physiol.

[133] Bhatt P, Gangola S, Chaudhary P, Khati P, Kumar G, Sharma A, Srivastava A (2019). Pesticide induced up-regulation of esterase and aldehyde dehydrogenase in indigenous *Bacillus* spp. Bioremediat J.

[134] Bustamante M, Duran N, Diez MC (2012). Biosurfactants are useful tools for the bioremediation of contaminated soil: a review. J Soil Sci Plant Nutr.

[135] Bhatt P, Huang Y, Rene ER, Kumar AJ, Chen S (2020). Mechanism of allethrin biodegradation by a newly isolated *Sphingomonas trueperi* strain CW3 from wastewater sludge. Bioresour Technol.

[136] Bhatt P, Zhang W, Lin Z, Pang S, Huang Y, Chen S (2020). Biodegradation of allethrin by a novel fungus *Fusarium proliferatum* strain CF2, isolated from contaminated soils. Microorganisms.

[137] Bhatt P, Huang Y, Zhang W, Sharma A, Chen S (2021). Binding interaction of glyphosate with glyphosate oxidoreductase and C–P lyase: Molecular docking and molecular dynamics simulation studies. J Hazard Mater.

[138] Bhatt P, Zhou X, Huang Y, Zhang W, Chen S (2021). Characterization of the role of esterases in the biodegradation of organophosphate, carbamate, and pyrethroid pesticides. J Hazard Mater.

[139] Bhatt P, Rene ER, Kumar AJ, Zhang W, Chen S (2020). Binding interaction of allethrin with esterase: Bioremediation potential and mechanism. Bioresour Technol.

[140] Zhang W, Lin Z, Pang S, Bhatt P, Chen S (2020). Insights into the biodegradation of lindane (γ-hexacholocyclohexane) using a microbial system. Front Microbiol.

[141] Singh A, Van Hamme JD, Ward OP (2007). Surfactants in microbiology and biotechnology: Part 2. Application aspects. Biotechnol Adv.

[142] Noordman WH, Janssen DB (2002). Rhamnolipid stimulates uptake of hydrophobic compounds by *Pseudomonas aeruginosa*. Appl Environ Microbiol.

[143] Singh P, Saini HS, Raj M (2016). Rhamnolipid mediated enhanced degradation of chlorpyrifos by bacterial consortium in soil-water system. Ecotoxicol Environ Saf.

[144] Feng Y, Huang Y, Zhan H, Bhatt P, Chen S (2020). An overview of strobilurin fungicide degradation: Current status and future perspective. Front Microbiol.

[145] Mohanty S, Mukherji S (2012). Alteration in cell surface properties of *Burkholderia* spp. during surfactant-aided biodegradation of petroleum hydrocarbons. Appl Microbiol Biotechnol.

[146] Mata-Sandoval JC, Karns J, Torrents A (2002). Influence of rhamnolipids and triton X-100 on the desorption of pesticides from soils. Environ Sci Technol.

[147] Mulligan CN (2009). Recent advances in the environmental applications of biosurfactants. J Colloid Inter Sci.

[148] Guo Q, Yan J, Wen J, Hu Y, Chen Y, Wu W (2016). Rhamnolipid-enhanced aerobic biodegradation of triclosan (TCS) by indigenous microorganisms in water-sediment systems. Sci Total Environ.

[149] Mani KA, Hamid SS, Ramalingam S, Kaliamoorthi R (2011). Effect of rhamnolipid potential on biodegradation of endosulfan by *Pseudomonas aeruginosa* in batch studies. J Biosci Technol.

[150] Wang B, Wang Q, Liu W, Liu X, Hou J, Teng Y, Luo Y, Christie P (2017). Biosurfactant-producing microorganism *Pseudomonas* sp. SB assists the phytoremediation of DDT-contaminated soil by two grass species. Chemosphere.

[151] Purnomo AS, Ashari K, Hermansyah FT (2017). Evaluation of the synergistic effect of mixed cultures of white-rot fungus *Pleurotus ostreatus* and biosurfactant-producing bacteria on DDT biodegradation. J Microbiol Biotechnol.

[152] Odukkathil G, Vasudevan N (2015). Biodegradation of endosulfan isomers and its metabolite endosulfate by two biosurfactant producing bacterial strains of *Bordetella petrii*. J Environ Sci Health B.

[153] Odukkathil G, Vasudevan N (2016). Residues of endosulfan in surface and subsurface agricultural soil and its bioremediation. J Environ Manag.

[154] Garcia Reyes S, Yanez Ocampo G, Wong Villarrealc A, Rajaretinam RK, Thavasimuthue C, Patiñof R, Ortiz Hernández ML (2017). Partial characterization of a biosurfactant extracted from *Pseudomonas* sp. B0406 that enhances the solubility of pesticides. Environ Technol.

[155] Gaur VK, Bajaj A, Regar RK, Kamthan M, Jha RR, Srivastava JK, Manickam N (2018). Rhamnolipid from a *Lysinibacillus sphaericus* strain IITR51 and its potential application for dissolution of hydrophobic pesticides. Bioresour Technol.

[156] Manickam N, Bajaj A, Saini HS, Shanker R (2012). Surfactant mediated enhanced biodegradation of hexachlorocyclohexane (HCH) isomers by *Sphingomonas* sp. NM05. Biodegradation.

[157] Bacerra-Castro C, Kidd PS, Rodriguez-Garrido B, Monterroso C, Santos-Ucha P, Prieto-Fernandez A (2013). Phytoremediation of hexachlorocyclohexane (HCH) contaminated soil using *Cytisus striatus* and bacterial inoculants in soil with distinct organic matter content. Environ Pollut.

[158] Abdul Salam J, Das N (2013). Enhanced biodegradation of lindane using oil-in-water bio-microemulsion stabilized by biosurfactant produced by a new yeast strain, *Pseudozyma* VITJzN01. J Microbiol Biotechnol.

[159] Nair AM, Rebello S, Rishad KS, Asok AK, Jisha MS (2015). Biosurfactant facilitated biodegradation of quinalphos at high concentrations by *Pseudomonas aeruginosa* Q10. Soil Sedim Contam Int J.

[160] Anu Appaiah KA, Karanth NGK (1991). Insecticide specific emulsifier production by hexachlorocyclohexane utilizing *Pseudomonas tralucida* Ptm^+^ strain. Biotechnol Lett.

[161] Bhatt P, Verma A, Verma S, Anwar MS, Prasher P, Mudila H, Chen S (2020). Understanding phytomicrobiome: A potential reservoir for better crop management. Sustainability.

[162] Banerjee S, Duttagupta S, Chakrabarty AM (1983). Production of emulsifying agent during growth of *Pseudomonas cepacia* with 2,4,5-trichlorophenoxyacetic acid. Arch Microbiol.

[163] Zenginyurek O. Effects of biosurfactants on remediation of soils contaminated with pesticides. Master’s thesis, *Izmir Institute of Technology*. 2002.

[164] Jakinala P, Lingampally N, Kyama A, Hameeda B (2019). Enhancement of atrazine biodegradation by marine isolate *Bacillus velezensis* MHNK1 in presence of surfactin lipopeptide. Ecotoxicol Environ Saf.

[165] Wawrzynczyk J, Szewczyka E, Norrlow O, Szwajcer Dey E (2007). Application of enzymes, sodium tripolyphosphate and cation exchange resin for the release of extracellular polymeric substances from sewage sludge characterization of the extracted polysaccharides/glycoconjugates by a panel of lectins. J Biotech.

[166] Liu Y, Fang HHP (2003). Influences of extracellular polymeric substances (EPS) on flocculation, settling, and dewatering of activated sludge. Crit Rev Environ Sci Technol.

[167] Sesay ML, Ozcengiz G, Sanin FD (2006). Enzymatic extraction of activated sludge extracellular polymers and implications on bioflocculation. Water Res.

[168] Dey ES, Szewczyk E, Wawrzynczyk J, Norrlow O (2006). A novel approach for characterization of exopolymeric material in sewage sludge. J Res Sci Technol.

[169] Mulligan CN (2005). Environmental Applications for Biosurfactants. Environ Pollut.

[170] Soberón-Chávez G, Maier RM, Soberón-Chávez G (2011). Biosurfactants: a General Overview. Biosurfactants.

[171] Akbari S, Abdurahman NH, Yunus RM, Fayaz F, Alara OR (2018). Biosurfactants-a new frontier for social and environmental safety: A mini review. Biotech Res Inno.

[172] Zhang HZ, Long XW, Sha RY, Zhang GL, Meng Q (2009). Biotreatment of oily wastewater by rhamnolipids in aerated active sludge system. J Zhejiang Univ Sci B.

[173] Li JQ, Liu WZ, Cai WW, Wang B, Ajibade FO, Zhang ZJ, Tiang XD, Wang AJ (2019). Applying rhamnolipid to enhance hydrolysis and acidogenesis of waste activated sludge: Retarded methanogenic community evolution and methane production. RSC Adv.

[174] White D, Hird L, Ali S (2013). Production and characterization of a trehalolipid biosurfactant produced by the novel marine bacterium *Rhodococcus* sp., strain PML026. J Appl Microbiol.

[175] Ndlovu T, Khan S, Khan W (2016). Distribution and diversity of biosurfactant-producing bacteria in a wastewater treatment plant. Environ Sci Pollut Res.

[176] Fang Y, Hozalski RM, Clapp LW, Novak PJ, Semmens MJ (2002). Passive dissolution of hydrogen gas into groundwater using hollow-fiber membranes. Water Res.

[177] Bhatt P, Bhatt K, Sharma A, Zhang W, Mishra S, Chen S (2021). Biotechnological basis of microbial consortia for the removal of pesticides from the environment. Crit Rev Biotechnol.

[178] Thavasi R, Jayalakshmi S, Balasubramanian T, Banat IM (2007). Biosurfactant production by *Corynebacterium kutscheri* from waste motor lubricant oil and peanut oil cake. Lett Appl Microbiol.

